# Assessment of Antioxidant and Cytoprotective Potential of Jatropha (*Jatropha curcas*) Grown in Southern Italy

**DOI:** 10.3390/ijms18030660

**Published:** 2017-03-18

**Authors:** Teresa Papalia, Davide Barreca, Maria Rosaria Panuccio

**Affiliations:** 1 Department of Agricultural Science, “Mediterranea” University, Feo di Vito, 89124 Reggio Calabria, Italy; tpapalia@unime.it (T.P.); mpanuccio@unirc.it (M.R.P.); 2 Department of Chemical, Biological, Pharmaceutical and Environmental Sciences, University of Messina, 98166 Messina, Italy

**Keywords:** *Jatropha curcas* L., RP-HPLC-DAD analysis of flavonoids, cytoprotective activities, antioxidant, polyphenols

## Abstract

Jatropha (*Jatropha curcas* L.) is a plant native of Central and South America, but widely distributed in the wild or semi-cultivated areas in Africa, India, and South East Asia. Although studies are available in literature on the polyphenolic content and bioactivity of *Jatropha curcas* L., no information is currently available on plants grown in pedoclimatic and soil conditions different from the autochthon regions. The aim of the present work was to characterize the antioxidant system developed by the plant under a new growing condition and to evaluate the polyphenol amount in a methanolic extract of leaves. Along with these analyses we have also tested the antioxidant and cytoprotective activities on lymphocytes. RP-HPLC-DAD analysis of flavonoids revealed a chromatographic profile dominated by the presence of flavone *C*-glucosydes. Vitexin is the most abundant identified compound followed by vicenin-2, stellarin-2, rhoifolin, and traces of isovitexin and isorhoifolin. Methanolic extract had high scavenging activity in all antioxidant assays tested and cytoprotective activity on lymphocytes exposed to tertz-buthylhydroperoxide. The results highlighted a well-defined mechanism of adaptation of the plant and a significant content of secondary metabolites with antioxidant properties, which are of interest for their potential uses, especially as a rich source of biologically active products.

## 1. Introduction

*Jatropha curcas* L. also known as physic nut (family *Euphorbiaceae*) can be classified as a large shrub or a small perennial tree able to reach a height between three and ten meters [1]. This plant is widespread in tropical and subtropical regions of Southeast Africa, Central and Latin America, Asia and India. *Jatropha curcas* L. is a species that is able to grow in dry and hot conditions, as, for instance, in fringe areas of semi-arid regions, where many species do not survive [2,3].

The result of adaptations to living in relatively harsh environmental conditions is a crop that is useful for the study of key physiological mechanisms adopted by plant to overcome multiple stresses [3].

The main interest for this plant is in regards to its great potential for biodiesel production. In fact, the high content of oil in *Jatropha curcas* L. seeds (up to 60% dependent on geographical and climatic conditions) can be used directly or in transesterified form as a biodiesel [4,5]. In addition, this plant is gaining a lot of attention because of its multipurpose and noteworthy economic potential [6]. The coagulant capacity, for instance, of industrial effluent obtained by grounded seeds is well known for the control of environmental pollution [7]. For centuries preparations of all parts of the plant (such as seed, leaf, stem bark, fruit, and latex) have found wide utilization in traditional medicine and for veterinary purposes. Detoxified oil of *Jatropha curcas* L. represents a rich protein supplement in animal feed [8]. In the literature, several biological effects were reported for the plant such as wound-healing, anti-inflammatory, antimalaria, antiparasitic, antimicrobial, insecticidal, antioxidant, and anticancer activity [9,10,11,12,13,14,15,16]. Literature data are available on the composition and biomedical applications of *Jatropha curcas* L. leaves and the identified compounds include cyclic triterpenes, alkaloids, and flavonoids [17]. The leaves were used as remedy for malaria, rheumatic, and muscular pains [18,19].

In vivo studies on antihyperglycemic activity of methanolic extract of leaves of *Jatropha curcas*. L were also reported [20]. Knnappan et al. [21], demonstrated the in vivo antiulcer activity of alcoholic extract of leaves. Furthermore, methanolic and aqueous extracts of leaves of *Jatropha curcas* L. have been found to inhibit drug-resistant HIV strains and hemagglutinin protein of influenza virus [22,23].

The present study is part of a research project, funded by the Calabria Region, aimed to promote the cultivation of *Jatropha curcas* L. in Calabrian marginal areas, for agriculture and bioenergy purposes. The considerable potential of this plant, the low input requirements, and its lower CO_2_ footprint in comparison with other oil-bearing crops, as well as the ability to prevent soil erosion problems, are the main advantages and the main reasons to promote *Jatropha curcas* L. cultivation in Calabrian marginal soils [24,25]. *Jatropha curcas* L. plants, originating from seeds of Kenyan trees were grown in hot and arid climatic conditions in Melito di Porto Salvo (Reggio Calabria, Italy) on a sandy-loam moderately alkaline soil. The objective was to evaluate phytochemical content and enzymatic mechanisms carried out by *Jatropha curcas* L. as strategies for its environmental adaptability. In order to improve the knowledge and to valorize this Calabrian population as a source of natural bioactive molecules, we have performed RP-HPLC-DAD analysis of a leaf methanol extract to evaluate polyphenol amount and, jointly, we have also tested antioxidant and cytoprotective activities on lymphocytes and erythrocyte membranes treated with tert-butylhydroperoxide (t-BOOH).

## 2. Results and Discussion

*Jatropha curcas* L. has a life expectancy of up to 50 years and is able to grow under a wide range of soil regimes (such as in deep, fertile, and loose soil), but it does not tolerate sticky, impermeable, and waterlogged soils. This plant requires sufficient sunshine, and cannot grow well under shade [2]. In this study we investigated how *Jatropha curcas* L. plants, originating from seeds of Kenyan trees, have adapted in Southern Italy, precisely in Melito Porto Salvo (Reggio Calabria). In this country the climate is warm, with an average temperature of about 18 °C and annual average rainfall of 767 mm. Chemical and physical characteristics of Melito soil evidenced a sandy-loam, moderately alkaline soil, with a low content of carbonates and a low salinity (Table 1). The amount and composition of soil organic matter (SOM) is strictly related to the performance of soil, in terms of quality and fertility, and a two percent SOM content (Table 1) is considered sufficient in these soils. The ratio of total organic carbon and total nitrogen (C/N ratio) is a traditional indicator to quantify the nature and the humification level of the organic matter present in soil. In general, in soils with a C/N ratio between 9 and 11, organic matter is well humified and quantitatively fairly stable over time. Results showed a C/N ratio lower than 9–10 indicating in Melito soil a prevalence of oxidation reactions leading to a decrease of the content of organic substance and in nitrogen release (Table 1).

### 2.1. Phytochemical Screening and Antioxidant Activity

In order to assess the degree of adaptation of *Jatropha curcas* L. plants located in Melito Porto Salvo, a phytochemical screening was performed. Since photosynthesis is one of the primary processes most affected by abiotic stresses [26,27], the evaluation of photosynthetic pigments and reactive oxygen species (ROS) content are considered traditional parameters to evaluate the performance and adaptation degree of a species. The high detected level of chlorophylls confirmed a good adaptation of plants in these soil and climatic conditions. ROS are generated as natural products of plant cellular photosynthetic and aerobic metabolism. Chloroplasts are a major site of ROS produced by energy transfer in photosynthetic electron transfer chains [28]. Peroxisomes and glyoxysomes also generate reactive oxygen species during metabolic pathways of photorespiration and fatty acid oxidation [29]. ROS have different roles in the organism and, at low concentration, for example, they behave as signal molecules for the activation/block of metabolic processes [30,31]. This mechanism of ROS homeostasis is maintained by enzymatic components such as superoxide dismutase (SOD), ascorbate peroxidase (APX), and catalase (CAT), and non-enzymatic compounds like ascorbic acid (ASA), reduced glutathione, a-tocopherol, carotenoids, phenolics, and flavonoids [32]. SODs are the only plant enzymes able to scavenge the superoxide anion. Moreover, in different cell compartments, Cat or APX (which utilize ascorbate as a reductant) eliminate H_2_O_2_ produced in the reaction catalyzed by SOD [33]. Catalase is unique among antioxidant enzymes in not requiring a reducing equivalent [34]. H_2_O_2_, being moderately reactive, does not cause extensive damage by itself; it can cross membranes and traverse considerable distance within the cell. At low concentration, H_2_O_2_ acts as regulatory signal for essential physiological processes, cell cycle, growth, and development [35]. Results on antioxidant enzymes showed significant modifications of dehydroascorbate reductase (DHA Rd), peroxidases (POX), and ascorbate peroxidase (APX) enzymes. Moreover, APX activity and ascorbate-glutathione cycle have a fundamental role in several cellular compartments such us peroxisomes, cytosol, chloroplasts, and mitochondria [33]. DHA Rd enzyme is responsible for the regeneration of ascorbic acid from an oxidized state in a reaction requiring glutathione. CAT activity in fresh leaves of *Jatropha curcas* L. was very low compared to other enzymes. However, CAT activity is generally low under normal growth conditions and it increases only at relatively high H_2_O_2_ concentrations or under stress conditions to support APX, SOD, and other peroxidases primarily involved in ROS homeostasis. The values obtained for APX and DHA Rd activities are in line with a high content of reduced glutathione and ascorbic acid detected in leaves of *Jatropha curcas* L. (Table 2). The amount of carotenoids, anthocyanins, and glutathione, and also a high ascorbic acid content with respect to the dehydroascorbic acid concentration (Table 2), indicate how the plant has developed its antioxidative defense system in the acclimation process for controlling ROS homeostasis. Plant phenolics constitute one of major groups of compounds acting as primary antioxidant or free radical terminators [32,36,37,38,39,40,41,42,43,44,45,46]. The leaf extracts of *Jatropha curcas* L. are also rich in phenolic compounds and tartaric acid ester derivatives (Table 2), which further contribute to the health promoting properties of this plant. The total amount of phenolic compounds are in line with the amounts detected in other water extracts of jatropha plants collected in different seasons [47], but are obviously inferior to the one obtained in organic solvent, where the total amount is notoriously higher than water extract [48,49,50,51]. The analyses of enzymatic and non-enzymatic antioxidants results show that in *Jatropha curcas* L. leaves there are remarkable amounts of these active components, allowing us to hypothesize a direct role in the ability of the plant to resist environmental stresses and improve survival potentiality in the new habitat.

### 2.2. Analysis of Anti-Peroxidative and Cytoprotective Activity

**Figure 1:**
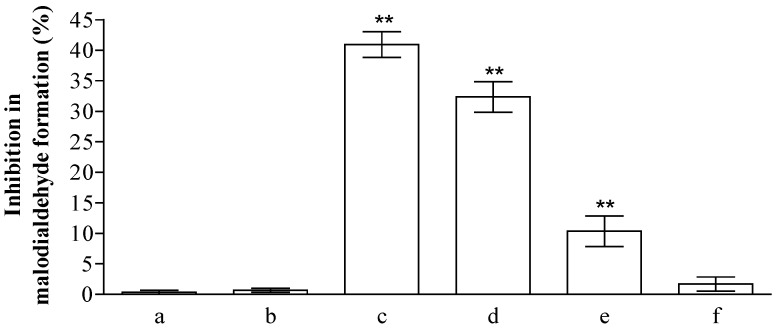
Inhibition (%) of erythrocyte membranes lipid peroxidation by *Jatropha curcas* L. methanol extract. Hemolysates plus 100 µM of tert-butylhydroperoxide (t-BOOH) were incubated for 30 min in the absence (a) or in the presence of 1.0, 0.5, 0.25, and 0.1 µM GAE (c–f). To check the possible influence of the solvent present in the extract, we incubated the hemolysates in the presence of the same amounts of methanol present in the samples (b). The values are expressed as mean ± SD (*n* = 3). The ** shows significant statistical differences (*p* < 0.05) with respect to erythrocyte membranes treated in the presence of only t-BOOH.

**Figure 2:**
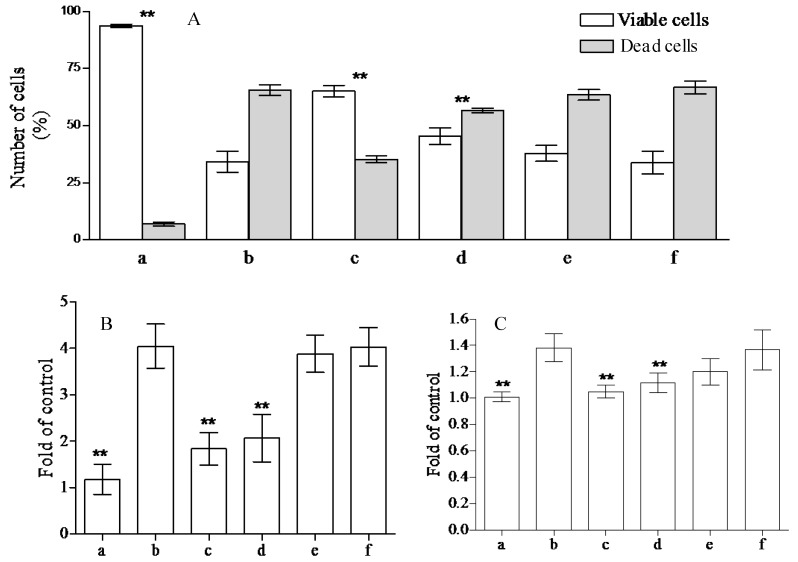
Cytoprotective effects of *Jatropha curcas* L. methanol extract on lymphocytes. Lymphocytes plus 100 µM of t-BOOH were incubated for 24 h in the absence (b) or in the presence of 1.0, 0.5, 0.25, and 0.1 µM GAE (c–f). To check the possible influence of the solvent present in the extract, we incubated the lymphocytes in the presence of the same amounts of methanol present in the samples (a). Cell vitality, integrity, and apoptotic events were analyzed by trypan blue staining (**A**); lactate dehydrogenase (LDH) release (**B**) and caspase 3 activation (**C**), respectively. The samples were analyzed by one-way ANOVA, followed by Tukey’s test. Asterisks ** indicate significant differences (*p* < 0.05) with respect to lymphocytes treated in the presence of only t-BOOH. Each value represents mean ± SD (*n* = 3).

The health promoting properties of the compounds present in the methanol extract were also analyzed to check their anti-peroxidative and cytoprotective ability on erythrocyte membranes and lymphocytes treated with tert-butylhydroperoxide (t-BOOH). Erythrocyte membrane lipid peroxidation has been performed by TBARS assay, analyzing the amount of malondialdehyde formation. t-BOOH (100 µM) is able to induce a remarkable amount of damage corresponding to the formation of ~1.22 ± 0.1 µM of malondialdehyde. The compounds present in the methanol extract of jatropha are able to reduce ~40, 33, 10, and 1% the formation of this compound utilizing 1.0, 0.5, 025, and 0.1 µM gallic acid equivalents (GAE), respectively (Figure 1). This activity is most probably due to the flavanone structure identified by chromatographic separation and in particular to the presence of apigenin derivatives. These results have been further supported by the analysis of cytoprotective activity of the extract against lymphocyte- t-BOOH treatment. A preliminary evaluation, obtained via incubating the cells with the same final gallic acid equivalent of methanol extract utilized in our work, shows no effects on lymphocytes (data not shown). As can be seen in Figure 2, the incubation of lymphocytes for 24 h at 37 °C in the presence of this strong oxidant (100 µM) induced a decrease of cellular vitality by up to 62%. The presence of methanol extract (at the final concentration of 1.0 and 0.5 µM GAE) remarkably improved cell survival with an increase of viable cells by ~1.9 and 1.3-fold, respectively, following treatment with t-BOOH. It was observed that 0.25–0.1 µM GAE have few or no effects on the process, resulting in values almost completely superimposable to the one obtained with cells incubated in the presence of only t-BOOH in the case of 0.1 µM GAE. The cytoprotective effects of the compounds present in the extract have been further analyzed taking into account the release of lactate dehydrogenase (LDH) from lymphocytes and the inhibition of caspase 3 activation. As can be seen in Figure 2, we highlight a decrease in the amount of LDH released in the samples incubated with t-BOOH in the presence of 1.0 and 0.5 µM GAE of methanol extract, as well as in the activation of caspase 3 in the same samples. Lower concentrations (0.25 and 0.1 µM GAE) were not able to induce statistically significant changes in the two enzymes analyzed. LDH is a marker of cell survival and compound toxicity due to its release outside the cells upon membrane damage, while caspases are one of the main markers of apoptosis onset. The decrease in the LDH release supports the hypothesis that the compounds present in the extract can directly act on t-BOOH by decreasing its strong oxidant activity, well evident at level of fatty acids peroxidation, and scavenging the reactive species that originated at the membrane level. This action is further confirmed by the process of caspase 3 activation, where the elimination of reactive species cannot be the trigger for its activation.

### 2.3. RP-DAD-HPLC Separation and Identification of Flavonoids Derivatives

**Figure 3:**
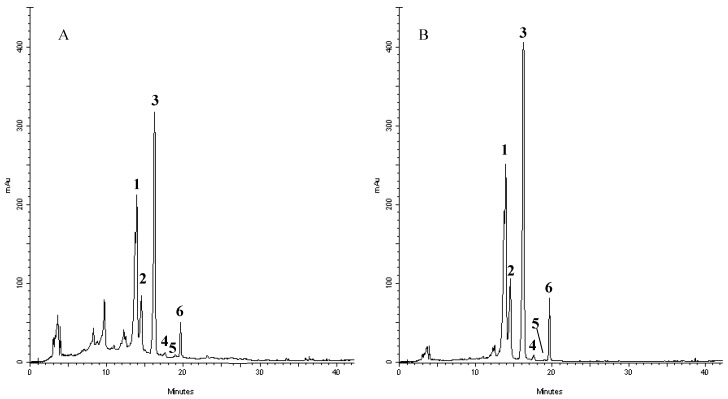
Representative HPLC chromatograms of flavonoids derivatives of *Jatropha curcas* L. methanol extract: absorbance at 278 nm (**A**) and 325 nm (**B**). Peak identification was performed by matching retention time and UV spectra against commercially available reference compounds. Peaks: vicenin-2 (**1**); stellarin-2 (**2**); vitexin (**3**); isovitexin (**4**); isorhoifolin (**5**); and rhoifolin (**6**).

In order to shed some light on the compounds that are present in the extract and responsible of such activities, we have performed a RP-DAD-HPLC separation to identify the presence of flavone and flavanone derivatives. The methanol extract was characterized by the presence of several well defined peaks belonging to flavonoids, as shown in the chromatograms recorded at 280 and 325 nm (Figure 3). This first approach let us to perform a preliminary screening based on the intense absorptions in the 270–280 nm region (Band II) of flavanone derivatives and the absorbance at the 320–330 nm region (Band I) where, principally, flavones and flavonols have remarkable absorption. The analyses of UV/visible spectrum of each peak show the presence of only flavone derivatives. Moreover, the identification of the compounds has been performed by means of acid hydrolysis and subsequent analysis of the aglycones and sugars. The chromatogram recorded for crude extracts after acidic hydrolysis (not shown) revealed that compounds 1–4 were resistant to HCl treatment, whereas 5–6 were hydrolyzed, providing evidence that the former flavonoids possessed C-linked saccharide moieties, whereas the latter bear O-linked glycosyl substituents. Moreover, the presence of a single aglycon molecule in the chromatograms revealed a pattern characterized by the presence of apigenin derivatives with the presence of glucose and rhamnose. The characteristic UV spectra, their retention time and co-elution with authentication standard let us to identify compounds as vicenin-2 (**1**); stellarin-2 (**2**); vitexin (**3**); isovitexin (**4**); isorhoifolin (**5**); and rhoifolin (**6**). Several of these compounds have already been reported in other leaves of *Jatropha curcas* L. (although grown in conditions different from the one tested in our experiment) and Jatropha genus suggesting a common pattern of flavonoids that are conserved in the species and may represent an indication of the endogenous adaptation of the plant to Calabrian marginal areas [17,52,53,54,55,56,57,58].

The quantifications of the identified flavonoids are depicted in Table 3.

### 2.4. Antioxidant Capacity

**Figure 4:**
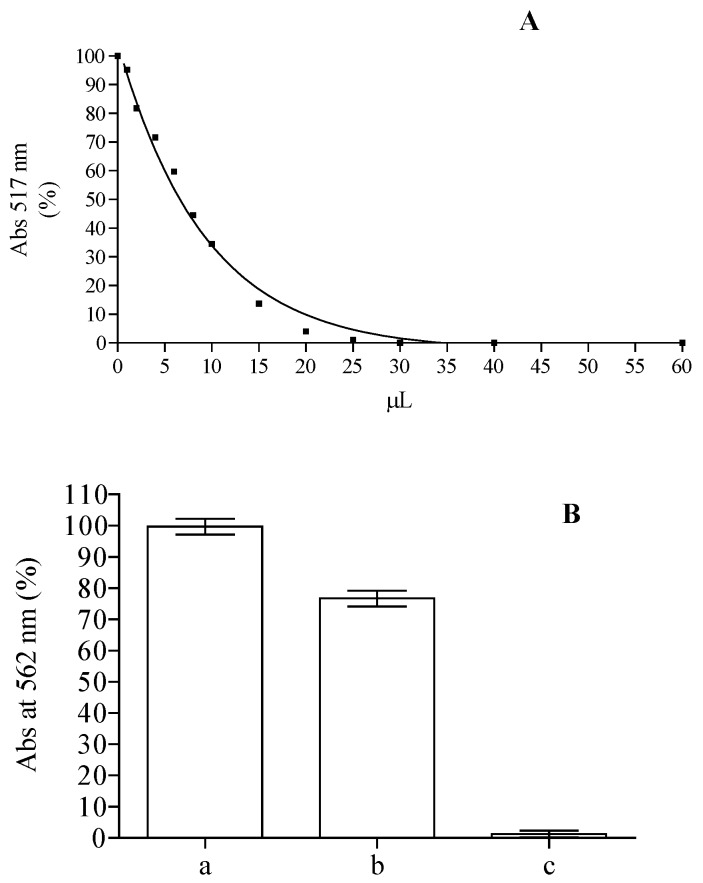
DPPH (**A**) and ferrozine assay (**B**) obtained with different amounts of *Jatropha curcas* L. methanol extract of *Jatropha curcas* L. leaves. Ferrozine assay without (a) or with 10 µL of methanol extract (b) or EDTA (c).

On the basis of the remarkable content of flavonoids and the presence of substituted flavone structures in the methanolic extract obtained from the leaves, we performed an in vitro biological assay in order to evaluate the antioxidant (DPPH, ABTS, FRAP, Ferrozine assay) and the cytoprotective activity of this extract. The DPPH is a stable radical frequently used to examine radical scavenging activity of natural compounds, and it is one of the starting points to check propensity of compounds or extracts to react with radicals. It has a strong absorbance at 517 nm due to its unpaired electron, giving the radical a purple color. Upon reduction with an antioxidant, its absorption decreases due to the formation of its non-radical form, DPPH-H. The activities of crude methanolic extracts in the scavenging of DPPH radical were concentration dependent. For instance (Figure 4A), the samples with 10 µL of methanol extract were able to reach up to ~65% of inhibition, corresponding to 14.7 µM trolox equivalents (TE). These results (IC_50_ = 58.8 TE µg/mL) are in line with the one obtained from ethanolic extract from the leaves of plants grown in Java, inferior to the ones obtained from the methanolic extract of leaves collected from plants grown in Malaysia and Egypt, but higher than the one obtained with plants grown in Iraq [48,49,50,59]. According to the ability of compounds present in methanolic extract to scavenge DPPH, ABTS radical formation was also inhibited with an activity corresponding to 16.25 ± 0.68 µM TE. These results suggest that the methanolic extract of *Jatropha curcas* L. leaves contains compounds capable of donating hydrogen to a free radical to eliminate its reactivity. Iron has a pivotal role in the wellness of organisms and it is also one of the main elements involved in the formation of radical species, so we tested the capability of the compounds present in the methanol extract while maintaining it in ferrous state and chelating it. The Fe^3+^–Fe^2+^ reducing power method is usually used in the determination of reducing power. The amount of Fe^2+^ can be determined by measuring the generation of Perl’s Prussian blue at 593 nm. The reducing power of 2.5 µL of the extract corresponds to 15.48 ± 2.9 µM of ascorbic acid equivalent. The chelating power of the extract was also tested using the ferrozine assay. Free transition metals can give rise to the generation of several ROS, in living organisms, through the oxidation of lipids, proteins and genetic materials; The presence of chelating agents can help organisms to stabilize and decrease the reactivity of these elements. As can be seen in Figure 4B, the decrease of the ferrozine–Fe^2+^ complex is influenced by the presence of the extract, although its activity is clearly lower than that of ethylenediaminetetraacetic acid (EDTA) utilized as positive control able to chelate all the ferrous present in the solution. The calculation of Oxygen Radical Absorbance Capacity (ORAC), utilizing a calibration curve obtained with trolox, showed a value of 7.71 ± 0.68 µmol TE/mg. This value is comparable to the one obtained for acetate, ethanol, and water extracts of *Jatropha curcas* L. seed shell [60].

By four different methods of antioxidant activity determination, we can see that the extract of *J. curcas* leaves exhibited relatively strong antioxidant activities, which may be due, at least in part, to their high phenolic content. In particular, the flavone derivatives characterized by the presence of a double bond at the 2, 3 position of the C ring conjugated with the 4-oxo group in position 4 may have a pivotal role in the process [41,42,43,44]. Recent studies indicate that all parts of this plant are valuable for multiple purposes, improving its valorization for large-scale plantation.

## 3. Materials and Methods

### 3.1. Reagents, Chemicals, and Instrumentation

HPLC-grade acetonitrile and methanol, as well as vicenin-2, vitexin, isovitexin, roipholin, isorhoifolin, and apigenin, were supplied by Sigma-Aldrich (St. Louis, MO, USA), while dimethylformamide (DMF) was supplied by Carlo Erba (Milano, Italy). All the other reagents and chemicals used in this study were of analytical grade and were purchased from Sigma (Sigma-Aldrich GmbH, Sternheim, Germany).

### 3.2. Chlorophyll and Carotenoid Pigments

Fresh leaves (0.050 g) were mixed with 2.5 mL of 100% ethanol in the dark for 24 h at 4 °C. Upon the conclusion of the incubation time the samples were centrifuged for 10 min at 7000 rpm. Lichtenthaler’s equation was employed to analyze the concentration of chlorophyll and carotenoid, based on absorbance at 649, 665, and 470 nm.

### 3.3. Anthocyanins

Fresh leaves (0.02 g) were extracted with 0.5 mL of a methanol:HCl solution (99:1, *v:v*) and centrifuged at 4 °C for 10 min at 7000 rpm. The absorbance of the supernatant was recorded at 530 and 657 nm and anthocyanin concentration was calculated according to the Equation (1):

[*A*_530 nm_ − (0.025 × *A*_657 nm_) × mL extract]/g fresh weight (1)

### 3.4. Tartaric Acid Esters and Total Phenols

Tartaric acid esters were tested by monitoring the absorbance change at 320 nm based on the procedure described by Romani et al. [61]. Fresh leaves (0.5 g) were extracted with 2 mL of methanol and centrifuged at 4 °C for 15 at 14,000 *g*. An aliquot of 25 µL of supernatant was diluted with 225 µL of 10% ethanol and 250 µL of 0.1% HCl in 95% ethanol, and 1 µL of 2% HCl was then added. The solution was mixed and tartaric acid ester were calculated at 320 nm as micrograms of caffeic acid/g fresh weight.

Total phenolic compounds have been analyzed by the Folin–Ciocalteu colorimetric method based on the procedure of Singleton et al. [62]. Dry leaves were extracted in water and the absorbance was recorded against blank at 765 nm and total phenols were expressed as mg tannic acid/g dry weight.

### 3.5. Reduced Glutathione

Reduced glutathione (GSH) level was determined by the method described by Jollow et al. [63]. Fresh leaf (0.5 g) homogenates in 3% of trichloroacetic acid were centrifugated at 3000 rpm at 4 °C. The supernatant was mixed with Ellman’s reagent and the absorbance of supernatant recorded at 412 nm and related to a calibration curve of GSH solutions (0–500 µg/mL).

### 3.6. Ascorbic and Dehydroascorbic Acid

Fresh leaves (0.5 g) were extracted in a chilled mortar with 5% metaphosphoric acid at 4 °C. After centrifugation at 18,000 rpm at 4 °C the supernatant was used for the determination of dehydroascorbic acid (DHA) and ascorbic acid (ASC) according to Law et al. [64].

### 3.7. Enzyme Assays

Fresh leaves were ground using a chilled mortar and pestle and homogenized in 0.1 M phosphate buffer solution (pH 7.0) containing 100 mg soluble polyvinylpolypyrrolidone (PVPP) and 0.1 mM ethylenediaminetetraacetic acid (EDTA). The homogenate was filtered through two layers of muslin cloth and centrifuged at 10,000 rpm for 20 min at 4 °C. The resulting supernatant was used for all assays.

Catalase (CAT, EC 1.11.1.6). The disappearance of H_2_O_2_ at 240 nm was determined according to Beaumont et al. [65] by using extinction coefficient (ε) = 0.036 mM^−1^·cm^−1^. The reaction mixture contained 1 mL potassium phosphate buffer (50 mM, pH 7.0), 40 µL enzyme extract, and 5 µL H_2_O_2_.

Peroxidase (POX, EC 1.11.1.7). The reduction in guaiacol concentration was determined by reading the absorbance at 436 nm continuously for 90 s. POX activity was quantified by the amount of tetraguaiacol formed using its extinction coefficient (ε) = 25.5 mM^−1^·cm^−1^ according to Panda et al. [66].

Dehydroascorbate reductase (DHA-Rd, EC 1.8.5.1). The reaction mixture contained 0.1 M K-phosphate buffer pH 6.5, 1 mM GSH, and 1 mM DHA. The activity was assayed following the increase in absorbance at 265 nm due to the production of ASC [67].

Ascorbate peroxidase (APX, EC 1.11.1.11). The decrease in absorbance at 290 nm, due to oxidation of ascorbate was determined according to Amako et al. [68]. The reaction mixture was 0.1 M K-phosphate buffer pH 6.5, 90 mM H_2_O_2_, and 50 mM ascorbate. Absorbance was recorded continuously for 90 s and APX activity was quantified by using the extinction coefficient, 14 mM^−1^·cm^−1^.

### 3.8. Preparation of Methanol Extract

The fresh leaves of *Jatropha curcas* L., harvested in summer 2013, were frozen at −20 °C. The frozen leaves were ground to a powder with a frozen mortar and ~10.0 g were extracted at room temperature under continuous stirring for 6 h with methanol (1:20 *w*:*v*). The samples were then centrifugated at 2500 rpm for 10 min and the supernatants were filtered with filter paper and evaporated to dryness in a rotavapor. This procedure was repeated three times and the powders obtained were resuspended in methanol to obtain a *w*:*v* ratio with the starting fresh leaves material of 1:1, with the end product utilized for RP-HPLC-DAD separation, antioxidant, and cytoprotective assays.

### 3.9. DPPH Radical Scavenging Assay

The antioxidant activity against 2,2-diphenyl-1-picrylhydrazyl DPPH radical was performed according to Molineux [69]. The assays were carried out by adding fixed amounts of extracts (0–60 µL) with DPPH solution (80 µM), resulting in the final volume of 1.0 mL. The reaction mixture was incubated for 30 min at 37 °C and, upon finishing the incubation time, the absorbance changes were recorded at 517 nm. The decrease in absorbance in percentage was analyzed utilizing the following equation:

Absorbance decrease (%) = 100 × (*A*_c_ − *A*_s_)/*A*_c_ (2)

where *A*_c_ is the absorbance of the control and *A*_s_ is the absorbance of the sample. Results have been expressed as Trolox equivalent (TE).

### 3.10. ABTS Radical Scavenging Assay

The 2,2'-azino-bis(3-ethylbenzothiazoline-6-sulphonic acid ABTS free radical-scavenging activity was carried out by a decolorization assay according to Re et al. [70]. Fixed amounts of the samples were added with the radical cation ABTS^+^ and the absorbance changes at 734 nm were recorded in a spectrophotometer after 6 min. The activity was expressed as inhibition in percentage at 734 nm using Trolox (1.1, 1.7, 2.3, 2.9, 3.5 µg/mL) as the reference compound.

### 3.11. Ferric-Reducing Antioxidant Power (FRAP) Assay

The ferric reducing antioxidant power assay was performed according to the method described by Benzie and Strain [71]. The samples were repeated in triplicate and the absorbance recorded at 593 nm after 4 min incubation at 37 °C. The antioxidant abilities of the extracts were expressed as equivalents of ascorbic acid utilizing a calibration curve obtained with fresh solutions of known ascorbic acid concentrations (0.005–0.02 mM).

### 3.12. Ferrozine Assay

The potential chelating activity of the extracts toward ferrous ions was analyzed by the method of Dorman et al. [72] with little modifications. As a reference compound we utilized EDTA (0.1 mM final concentration). The activity of the extract was performed by adding 10 µL to a solution of 0.5 mM FeSO_4_ (0.01 mL). After the addition of 5.0 mM ferrozine (0.4 mL) solution, the samples were shaken and left for 10 min at room temperature (RT). Finally, the absorbance at 562 nm was recorded with a spectrophotometer. The inhibition (%) of ferrozine Fe^2+^ complex formation was obtained using the following equation:

% Inhibition = [(*A*_c_ − *A*_s_)/*A*_c_] × 100 (3)

where *A*_c_ is the absorbance of the control and *A*_s_ is the absorbance of the samples in the presence of the extracts.

### 3.13. Flavonoids Profile Identification

The identification of flavonoids present in the methanol extract was performed by utilizing a Shimazu Reverse Phase–Diode Array Detection–High Performance Liquid Chromatography (Shimadzu, Kyoto, Japan) with injection loop of 20 µL. The column was a BioDiscovery C18 (Supelco, Bellefonte, PA, USA) of 250 mm × 4.6 mm i.d., 5 µm and equipped with a 20 mm × 4.0 mm guard column. The temperature was set at 30 °C and flow-rate at 1.0 mL/min. The separation was performed utilizing a linear gradient of acetonitrile in H_2_O as mobile phase. The gradient was: 0–15 min (5%–20% of acenitrile), 15–20 min (20%–30% of acetonitrile), 20–35 min (30%–100% of acetonitrile), 35–40 min (100% of acetonitrile), 40–45 min (100%–5% of acetonitrile), and 45–55 min (5% of acetonitrile). The chromatograms were recorded at 278 and 325 nm and UV/visible spectra of each peak were between 200 and 450 nm. The identification of the compounds were performed according to retention time, UV spectra, and co-elution with authentication standards. Quantitative analysis was carried out by integration of the areas of the peaks from the chromatogram at 325 nm and comparison with calibration curves obtained with the known concentration of a commercially available standard (0.1–10 mg/L).

### 3.14. Acid Hydrolysis

Acid hydrolysis of the samples was performed according to Hertog et al. [73].

### 3.15. Erythrocytes Lipid Peroxidation Assay

Hemolysates were prepared according to Barreca et al. [38] and lipid peroxidation was analyzed by thiobarbituric acid reactive substance (TBARS) assay [74].

### 3.16. Lymphocyte Isolation

Lymphocytes were isolated according to Barreca et al. [42,44,45] and utilized in the following tests.

### 3.17. Cytotoxicity Assays

To perform the cytotoxicity assay, we treated cells (1 × 10^6^/mL) with 100 µM of t-BOOH in the absence or in the presence of 1.0, 0.5, 0.25, and 0.1 µM gallic acid equivalents (GAE) of the extracts for 24 h. Parallel controls were performed without t-BOOH, but in the presence of the same final gallic acid equivalents of methanol extract utilized during experimentation. Moreover, in all experiments blanks, without t-BOOH, were performed [38,75]. The cell viability, after finishing the incubation period were established with trypan blue staining. The cells were diluted 1:1 (*v:v*) with 0.4% trypan blue and counted with an haemocytometer. Results are expressed as the percentage of live or dead cells (ratio of unstained or stained cells to the total number of cells, respectively). To check cytotoxicity we also analyzed lactate dehydrogenase (LDH) release from damaged cells into culture medium with a commercially available kit from BioSystems (Barcelona, Spain). Extracts did not show interference with the determination of LDH at the concentration utilized in the experiments. For caspase activity determination, we followed the procedures described by Bellocco et al. [45].

### 3.18. Oxygen Radical Absorbance Capacity (ORAC) Assay

The ORAC assay was performed according to Dàvalos et al. [76] with few modifications. Twenty microliters of methanol extract were added to 120 µL of fresh fluorescein solution (117 nM). After a preincubation time of 15 min at 37 °C, we added 60 µL of freshly prepared 2,2′-Azobis(2-methylpropionamidine)dihydrochloride (AAPH) solution (40 mM). Fluorescence was recorded every 30 s for 90 min (*λ*_ex_: 485; *λ*_em_: 520). A blank using 20 µL of methanol instead of the sample was also analyzed, along with a reference calibration curve with Trolox (10–100 µM). The ORAC value was expressed as µmoles of Trolox Equivalent (TE)/mg of fresh weight (F.W.) sample. All assays were carried out in triplicate.

### 3.19. Statistical Analysis

The values of the data are expressed as means ± standard deviation. One-way analysis of variance (ANOVA) was performed on the obtained results. Tukey’s test was run to check the significance of the difference between the samples and the respective controls. A *p* < 0.05 value indicates statistically significant difference.

## 4. Conclusions

In this study the obtained results concerning a phytochemical and enzymatic screening suggested that the *Jatropha curcas* L. plants, originated from Kenya and grown in Melito Porto Salvo, are well suited to the typical Mediterranean climate of Southern Italy. Moreover, the methanolic extract of the leaves shows very interesting antioxidant and cytoprotective activities, which can be attributed also to its flavonoids profile, which is dominated by the presence of flavone compounds, one of the most studied and promising forms of secondary metabolites for potential use as nutraceuticals. Therefore, methanolic extracts of *Jatropha curcas* L. leaves could represent a promising source of natural antioxidants compounds to employ in the pharmaceutical and cosmetic industries.

## Figures and Tables

**Table 1:** Chemical and physical characteristics of field for *Jatropha curcas* L. cultivation.

Texture	pH	E.C. (mS/cm)	Total Carbonates (%)	TOC (%)	SOM (%)	N (g/kg)	C/N
Loam-sandy	8.20	1.65	2.00	14.06	2.60	1.82	7.73

Electrical conductivity (E.C.); Soil organic matter (SOM); total organic carbon (TOC); Nitrogen (N); ratio of total carbon and total nitrogen (C/N).

**Table 2:** Analysis of phytochemical composition and enzymatic antioxidants of leaves of *Jatropha curcas* L. Value were expressed as mean ± standard error (*n* = 3).

Phytochemical Screening of *Jatropha curcas* L. Leaf	Value
Chlorophyll a (mg·g^−1^ Fresh Weight)	1.60 ± 0.10
Chlorophyll b (mg·g^−1^ Fresh Weight)	0.90 ± 0.03
Catalase (CAT) activity (nmol H_2_O_2_·g^−1^ Fresh Weight)	14.75 ± 1.20
Peroxidases (POX) activity (µmol guaiacol·g^−1^ Fresh Weight)	1.06 ± 0.04
Ascorbate peroxidase (APX) activity (µmol H_2_O_2_·g^−1^ Fresh Weight)	1.30 ± 0.04
Dehydroascorbate reductase (DHA-Rd) activity (µmol ASA·g^−1^ Fresh Weight)	0.77 ± 7.10
Ascorbic acid (ASA) (µmol ascorbic acid/g Dry Weight)	3.78 ± 0.19
Dehydroascorbic acid (µmol dehydroascorbic acid/g Dry Weight)	2.34 ± 0.20
Reduced glutathione (µmol GSH/g Dry Weight)	1.75 ± 0.14
Total phenols (mg tannic acid/g Dry Weight)	7.36 ± 0.60
Total carotenoids (mg/g Fresh Weight)	0.20 ± 0.03
Anthocyanins (µg anthocyanin·g^−1^ Fresh Weight)	9.42 ± 2.30
Tartaric acid esters derivatives (µg caffeic acid·g^−1^ Fresh Weight)	23.00 ± 0.10

**Table 3:** Flavonoids content in methanol extract of *Jatropha curcas* L. leaves.

Compounds	mg/kg F.W.
Vicenin-2	3.7 ± 0.41
Stellarin-2	1.2 ± 0.23
Vitexin	6.0 ± 0.52
Isovitexin	0.13 ± 0.04
Isorhoifolin	Trace
Rhoifolin	2.2 ± 0.25
